# Time‐limited diets and the gut microbiota in cardiometabolic disease

**DOI:** 10.1111/1753-0407.13288

**Published:** 2022-06-13

**Authors:** Karina Ratiner, Hagit Shapiro, Kim Goldenberg, Eran Elinav

**Affiliations:** ^1^ Systems Immunology Department Weizmann Institute of Science Rehovot Israel; ^2^ Microbiome & Cancer Division, DKFZ Heidelberg Germany

**Keywords:** cardiometabolic disease, circadian rhythms, gut microbiome, intermittent fasting, time‐restricted feeding, 肠道菌群, 心脏代谢性疾病, 间歇性禁食, 限时进食, 昼夜节律

## Abstract

In recent years, **intermittent fasting (IF)**, including periodic fasting and **time‐restricted feeding** **(TRF)**, has been increasingly suggested to constitute a promising treatment for **cardiometabolic diseases** **(CMD)**. A deliberate daily pause in food consumption influences the gut microbiome and the host circadian clock, resulting in improved cardiometabolic health. Understanding the molecular mechanisms by which circadian host‐microbiome interactions affect host metabolism and immunity may add a potentially important dimension to effective implementation of IF diets. In this review, we discuss emerging evidence potentially linking compositional and functional alterations of the gut microbiome with IF impacts on mammalian metabolism and risk of development of hypertension, **type 2 diabetes (T2D)**, obesity, and their long‐term micro‐ and macrovascular complications. We highlight the challenges and unknowns in causally linking diurnal bacterial signals with dietary cues and downstream metabolic consequences and means of harnessing these signals toward future microbiome integration into precision medicine.

## Graphical abstract

- Time‐specific diet is a novel nutritional approach to prevent and treat cardiometabolic disease.
- Feeding‐fasting cycles shape gut microbial composition and metabolite production. These alterations may play a causative role in driving the cardiometabolic benefits of intermittent fasting.
- Despite the impressive results in animals, human studies show contradicting results, possibly due to intraindividual variation involved in the host‐microbiome response to diet.
- Better mechanistic understanding is required to develop personalized dietary interventions to treat cardiometabolic diseases.

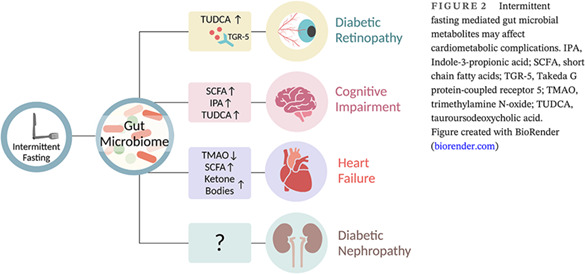

## 摘要

近年来, 间歇性禁食(IF), 包括周期性禁食和限时进食(TRF), 逐渐被认为是心脏代谢性疾病(CMDS)的潜在治疗方法。每天有意识地停止进食会影响肠道微生物群和宿主生物钟, 从而改善心脏代谢健康。了解昼夜宿主‐微生物组相互作用影响宿主新陈代谢和免疫的分子机制, 可能会为有效实施IF饮食增加一个潜在的重要维度。在这篇综述中, 我们讨论了IF可能改变肠道微生物组的组成和功能, 从而影响哺乳动物的新陈代谢以及高血压, 2型糖尿病(T2D), 肥胖及其长期微血管和大血管并发症的发展风险。我们强调了在将昼夜细菌信号与饮食线索和下游代谢进行因果关联的挑战和未知, 以及利用这些信号实现未来微生物组与精准医疗的整合的手段。

## 1 INTRODUCTION

**Cardiometabolic diseases (CMDs)**, mainly encompassing **cardiovascular diseases (CVDs)** and **type 2 diabetes** **(** **T2D** **)**, are rapidly increasing in prevalence worldwide.^1^, ^2^ In parallel, human eating behavior has dramatically changed over the past decades. The traditional breakfast‐lunch‐dinner pattern has been replaced by frequent snacking, large nighttime meals, and breakfast skipping.^3^, ^4^ Such irregular eating patterns may have an adverse effect on cardiometabolic risk factors, such as obesity, insulin resistance, and hyperglycemia,^4^ thus contributing to the rising prevalence of T2D.^1^ In contrast, intentional eating with awareness of the timing and frequency of eating occasions could improve lifestyle and cardiometabolic risk factor management,^5^ which can protect against the development of T2D and CVDs.^6^, ^7^

***Intermittent Fasting Regimens*.** **Intermittent fasting (IF)** is a general term for a predetermined pause in the consumption of food, usually accomplished by restricted eating for 12 hours to a few days per week.^5^ During this protocol, calories and macronutrients are consumed at precise times, followed by periods without food ingestion. There are different types of IF diets, ranging from relatively long fasting periods to those spanning only a few daily wake hours^5^ (Table 1). One of them is **alternate‐day fasting (ADF)**, which entails alternating between regularly eating one day and refraining from eating the next day. The feeding:fasting ratio of 5:2 days, also known as periodic fasting, is characterized by cycles of extreme limitation or complete abstinence of food for two days a week, whereas food can be eaten without restrictions on the other five days of the week. A variation of periodic fasting is the **fasting‐mimicking diet (FMD)**, which includes cycles of several consecutive days of low‐calorie intake.^8^ A less extreme diet is **time‐restricted feeding (TRF)**, in which individuals eat their meals within a particular window of time each day, for example, fasting:feeding of 12:12 hours or 16:8 hours, respectively. The flexibility in the fasting frequency and duration enables individuals to choose their preferential eating pattern. Within the TRF framework, the specific time of the day in which the food is consumed is of importance. Consuming calories during the active phase, in comparison with the resting phase, is typically associated with improved features of cardiometabolic health, probably affecting circadian rhythms.^5^, ^9^, ^10^, ^11^ Thus, it is advisable that within the TRF diet, calories should be consumed during the active phase. In obese mice fed with a high‐fat diet, restriction of feeding to the active phase (in the dark) led to reduced body weight and fat mass, improved glycemic control, reduced liver steatosis, and better running endurance compared with mice consuming the same calories and fed ad libitum.^12^ In line with these studies, some clinical trials suggest that eating breakfast may be associated with weight loss, improved glycemic control, and cardiovascular outcomes in humans.^4^, ^13^, ^14^ On the other hand, several randomized controlled trials demonstrated that eating breakfast does not significantly affect body fat composition or cardiometabolic parameters in humans compared to skipping breakfast and consuming an equivalent number of calories during a day.^15^, ^16^ Several clinical studies show that IF diets increase life expectancy and provide a wide range of benefits, including mitigation of obesity, hypertension, T2D, and CVDs.^17^, ^18^, ^19^ However, other studies have found that time‐specific diets are not superior to energy restriction in improving cardiovascular and metabolic outcomes.^20^, ^21^, ^22^, ^23^, ^24^ More recent meta‐analyses of randomized controlled trials in overweight or obese patients indicated that intermittent energy restriction involving 2–3 days of periodic fasting led to improved weight loss and reduced body fat versus continuous energy restriction.^25^, ^26^ In summary, there is a lack of agreement between clinical studies on whether IF may lead to improved metabolic outcomes. The conflicts between studies stem from differences in restricted feeding durations (eg, 12:12, 14:10, 16:8, ADF or periodic fasting), feeding time (during the active or resting phase), the amount of calories consumed during the feeding time, as well as the feeding regimens and the caloric content given to the control groups.^17^, ^20^, ^22^ In addition, body mass index, glycemic control, age, sex, physical activity, and geographic and ethnic differences between individuals had major implications on the metabolic outcomes of different IF diets.^17^, ^18^, ^20^, ^21^, ^23^, ^25^ With these limitations withstanding, it is possible that personalized, individually tailored time‐restricted diets will be more feasible, tolerable and sustainable, and will result in more consistent long‐term metabolic improvements.

**TABLE 1:** Types of intermittent fasting regimens

Fasting regimen	Description
Alternate‐day fasting (ADF)	Switching between a day of eating regularly followed by a day of fasting.
5:2 periodic fasting diet	Two days of fasting per week.
Fasting‐mimicking diet (FMD)	Several consecutive days of reduced caloric intake, followed by a normal eating cycle every one to four months or every other week. Most of the FMDs composition is based on plant‐derived compounds.
Time‐restricted feeding (TRF)	Limit daily food intake to a 4‐ to 12‐h window. This includes fasting during Ramadan.

There are several theories hypothesizing about the mechanisms potentially underlying the possible benefits of IF diets compared to ad libitum feeding. A critical factor in many of these benefits is the “metabolic switch,” a phenomenon in which fasting causes the body to preferentially use fatty acids and ketone bodies derived from fatty acids as a fuel instead of glucose.^27^ Consequently, ketone bodies can serve as signaling molecules that trigger response against oxidative and metabolic stress, as well as removal of damaged molecules.^18^, ^28^ Refeeding, on the other hand, results in increased levels of the incretin hormone **glucagon‐like peptide 1 (GLP‐1)** and insulin, which then enhance glucose uptake and promote tissue‐specific growth and plasticity.^18^, ^28^ There are ongoing efforts to identify the mechanisms by which the metabolic switch is controlled and influences the body. One of the hypotheses was that the biological clock is required for the TRF impact.^29^ However, it appears that TRF also improves the metabolic phenotype in mice with mutations in the biological clock genes.^29^ Thus, the mechanisms underlying the metabolic switch during fasting and feeding merits further investigation. More recently, IF has been shown to affect the gut bacteria,^30^, ^31^, ^32^, ^33^, ^34^, ^35^, ^36^ which are involved in virtually all aspects of host physiology, suggesting an entirely new mechanism for the physiological impact of IF.

***The gut microbiome*.** The digestive system of most living organisms is colonized by commensal bacteria, viruses, fungi, and parasites, collectively referred to as the gut microbiome. Overall, the gut microbiome consists of an enormous number of cells and functions, regarded by some as the equivalent of an additional organ.^37^ Dietary components provide ample substrates for microbial growth and production of bacterial metabolites, which in turn govern many aspects of host physiology, including metabolism, digestion, and immunity.^38^ Indeed, it has been shown that the intestinal microbiome has an effect on various diseases, including metabolic diseases,^39^, ^40^, ^41^ liver diseases,^42^, ^43^ neurodegenerative disorders,^44^, ^45^, ^46^, ^47^, ^48^ cancer,^49^ among others. As for metabolic disorders, multiple studies have suggested that obese individuals have a distinct profile of gut bacterial phyla compared to lean people, and in general obese compared to lean humans and rodents show lower bacterial diversity.^41^, ^50^, ^51^ Over the past decade, increasing evidence has emerged that the diversity, composition, and function of the gut microbiome are altered in individuals with cardiometabolic abnormalities.^52^, ^53^ Furthermore, CMD patients compared to healthy individuals feature differential microbial‐derived metabolites that can communicate with various remote organs and affect host physiology.^40^, ^41^, ^54^, ^55^ These include **short‐chain fatty acids** (**SCFAs**; eg, acetate, propionate, and butyrate), lactate, secondary bile acids, lipids and fatty acids, vitamins, flavonoids, byproducts from amino acids and protein metabolism, lipopolysaccharides, and **trimethylamine (TMA)**.^40^, ^41^, ^56^, ^57^, ^58^, ^59^, ^60^, ^61^, ^62^ The microbial compounds may promote health outcomes, depending on the dose of the particular metabolite as well as the specific phenotype being evaluated.^57^ For example, the bacterial‐derived secondary bile acids **hyodeoxycholate (HDCA)** and **lithocholate (LCA)** show a protective effect against infection by some clinically relevant *Clostridium difficile* strains,^63^ but the same concentrations of HDCA and LCA were found to be toxic to mammalian hepatic cells.^64^ Therefore, controlled quantity of a specific type of microbial metabolite is a critical task required for the host physiology. Through the activities of the gut microbiome, metabolites from food and environmental factors are converted into molecules that are precursors for the liver, which in turn form molecules that control the host physiological functions under various circumstances. For example, dimethylglycine and N‐acetylglycine jointly produced by the host and the gut microbiome from dietary choline can regulate weight gain after smoking cessation and may affect obesity beyond the smoking settings.^40^ All of these microbial‐associated metabolites can act as signaling molecules that affect the host physiology in metabolic health and disease. A causal role for the gut microbiome in CMDs progression has been demonstrated in studies using germ‐free mice that were colonized with microbes obtained from humans or rodents with cardiometabolic disorders, leading to the development of metabolic complications such as weight gain,^40^, ^41^, ^65^, ^66^ aberrant glycemic response,^67^, ^68^ and hypertension^69^ in recipient mice.

Because the gut microbiome shows a significant impact on the host physiology, it is of particular importance to understand which factors shape the gut microbiome or control its function. Dietary composition,^70^, ^71^ timing,^71^, ^72^, ^73^, ^74^ and duration^41^ affect the gut microbiome structure, which may persist even after diet discontinuation,^41^, ^75^ making dietary interventions a powerful tool for treating diseases. Recently, an increasing number of studies show that various IF diets alter the gut microbiome composition and function.^36^, ^69^, ^72^, ^73^, ^74^, ^76^, ^77^, ^78^, ^79^, ^80^, ^81^, ^82^ According to most reports, IF diets are usually accompanied by increased bacterial diversity, enhanced Firmicutes/Bacteroidetes ratio, and higher levels of SCFAs.^83^, ^84^, ^85^, ^86^ Past studies have related the Firmicutes/Bacteroidetes ratio to lower weight and improved cardiovascular health,^66^, ^87^, ^88^, ^89^ whereas some recent studies have shown conflicting results regarding the association of this bacterial ratio with improved metabolic outcomes.^90^ Additional compositional alterations induced by IF diets may include an increased abundance of species belonging to *Akkermansia* and *Lactobacillus* and decreased abundance of opportunistic pathogens, such as *Alistipes*, which were reported in lean and healthy humans and rodents.^34^, ^76^, ^91^ However, not all studies agree on the specific compositional alterations in bacterial communities following IF diet. For example, one study found a decrease in *Akkermansia* ^85^ in diabetic mice undergoing ADF, whereas another report showed no difference.^86^ In line with these observations, a specific phylum, such as Firmicutes or Bacteroidetes, may contain a different composition of bacterial species, thus changes reported at higher levels, especially at the phylum level, appears to be of limited value. Collectively, although different IF diets induce a prominent impact on the composition and function of the gut bacteria, the effect of this altered microbiome configuration on the host metabolic functions of the host requires more comprehensive studies.

***Host‐microbe circadian network*.** Rhythmic patterns exist at virtually all levels of biological organization and are considered one of the most important features of life. For example, mammals display daily rhythms in the form of sleep–wake cycles, cardiovascular activity, hepatic metabolism, and endocrine system function.^92^ A network of circadian clocks controls the timing of food intake by setting daily windows that overlap with the active phase of the host. Circadian clocks set the internal timing by synthesizing clock‐controlled proteins that send intracellular and eventually extracellular signals. These clock genes comprise the transcriptional factors *Bmal1* and *Clock*, whose protein products dimerize, enter the nucleus, and stimulate the transcription of repressor genes *Per1*, *Per2, Cry*, and *Nr1d1*.^93^, ^94^ The circadian clock is responsible for synchronizing metabolism and food intake with daily cycles^95^ and appears to influence microbial homeostasis. The microbiome exhibits daily oscillations, manifesting in dynamic compositional and functional profiles throughout the day derived from the host's eating behavior, which in turn stimulates oral^96^, ^97^, ^98^ and gut^72^, ^73^, ^74^, ^99^, ^100^, ^101^ microbial oscillations. Several studies using indirect perturbation of microbial rhythms or complete abolishment of the microbiome by antibiotics or germ‐free mice models revealed that the microbiome is responsible for the global circadian programming of the host transcriptome, epigenome, and metabolome.^73^, ^102^ In mice, absence of the microbiome alters rhythmic gene expression in multiple tissues, including colon, small intestine, liver, and adipose tissue.^73^, ^102^ Host‐microbiome circadian interactions are necessary for a variety of physiological processes, including metabolism and nutrient absorption,^103^, ^104^ energy expenditure,^105^ hepatic detoxification,^73^, ^106^ sexual growth,^102^ and immune function.^71^, ^102^, ^107^, ^108^ Observations in mice indicate that circadian programming is primarily derived from the diurnal penetration of commensal microbes through the mucus layer.^73^ The diurnal bacterial localization through feeding is likely involved in maintaining the ability of the host to perform various physiological functions, such as nutrient uptake and management of barrier integrity.^71^, ^108^

In rodents, a dampened gut microbiome oscillation was observed under high‐fat diet, jet lag, and in *Per1/2*‐deficient mice.^72^, ^73^, ^74^, ^99^ Under any of these conditions, mice also exhibit disruptions of biological clock gene expression and arrhythmic behavior and are subsequently prone to metabolic abnormalities.^109^, ^110^, ^111^ Interestingly, limiting the feeding time of these circadian‐disrupted mice with TRF alleviates their metabolic abnormalities^29^, ^112^ and also restores their gut microbiome rhythm.^72^, ^74^, ^84^ In line with these studies in rodents, it is possible that modulating the diurnal rhythms of the gut microbiome by IF diets could be developed in the future as a valuable tool to promote health and treat metabolic disorders in humans. However, it is challenging to generalize the findings from murine data to humans, because of a large intervariability between humans in clinical studies undergoing IF diets, variations in fasting:feeding duration and variations in the caloric content of the diet given to the participants and their corresponding control groups as mentioned previously. Another major difference between rodent and human studies results from the source of the microbial sampling. Because of accessibility considerations, most human studies are conducted on stool samples. Stool samples, however, do not represent mucosal microbes that have been linked to circadian programming.^73^ Nevertheless, a loss of diurnal rhythms in the gut microbiome obtained from stool samples has been reported in both humans and rodents.^85^, ^100^, ^113^ With respect to dietary patterns, it has been reported that IF restructured the stool microbiome of diabetic mice resulting in altered bacterial configuration compared to control mice.^85^ Therefore, despite possible discrepancies arising from limited information on mucosal‐associated bacteria in different parts of the gut, stool samples may still offer some valid information. Collectively, the role of the bacterial rhythmicity in cardiometabolic health and diseases should be further investigated.

## 2 INTERMITTENT FASTING AND MICROBIAL REGULATION IN CARDIOMETABOLIC DISORDERS

Fluctuations in food availability affect the entire ecosystem, including host and symbiotic communities, and have profound implications on cardiometabolic health. In this section, we highlight some of the remarkable discoveries about the microbiome's role in IF, which in turn influence metabolic manifestations related to CMDs such as obesity, hyperglycemia, and hypertension.

***Obesity and lipid metabolism*.** Fat accumulation in the body that causes obesity presents serious health risks to the cardiovascular system. Excessive energy intake coupled with lower energy expenditure leads to a weight gain, which is usually composed of accumulation of body fat.^114^, ^115^, ^116^ Rodents fed with high‐fat diets develop obesity and metabolic disorders and are widely used in research to mimic the human metabolic syndrome.^117^ In mice fed with high‐fat diet, TRF was found to reduce body weight and fat mass, independent of total caloric intake and without any change in physical activity.^12^ Meta‐analysis of randomized controlled trials in humans showed that intermittent energy restriction by periodic fasting of 2–3 days per week led to improved weight loss and lower body fat in overweight and obese subjects, in comparison with matched chronic calorie restriction,^25^, ^26^ but this was not the case for TRF or ADF diets^22^ or for lean participants subjected to the ADF regimen.^21^ A significant contribution to these improvements can be attributed to the impact of the gut microbiome on lipid metabolism and energy balance.^118^ One such mechanism involves adipose tissue, a heterogeneous organ sensitive to nutritional stimuli and undergoing dynamic remodeling during IF. Adipose tissues are found in two different forms in mammals: **white adipose tissues (WAT)** that store energy as triglycerides and brown adipose tissues that burn extra calories to generate heat.^119^ Within the WAT, beige adipocytes have similar properties to brown adipose tissue and are formed in response to various stimuli, predominantly cold temperatures.^120^ Li et al.^118^ reported that ADF modulates the formation of beige adipocytes through microbiome‐dependent mechanisms. In this study, obese mice fed with high‐fat diet and undergoing an ADF regimen exhibited an increased accumulation of beige fat in WAT accompanied by weight loss and altered gut microbiome composition.^118^ Notably, antibiotics supplementation to obese mice undergoing ADF abrogated the beneficial metabolic effects of ADF, and fecal transplantation from ADF mice to antibiotics‐treated obese mice improved metabolic health, suggesting a causal role for the gut microbiome in metabolic improvements mediated by ADF.^118^ Combining cecal metabolomics with shotgun metagenomics revealed that ADF induced alterations in the composition of the gut microbiome toward lactate‐ and acetate‐producing bacteria, such as *Lactobacillus reuteri*, which in turn resulted in accumulation of serum lactate and acetate. ADF diet also led to enhanced energy expenditure by promoting beige adipogenesis, and ameliorated weight gain and other metabolic disorders.^118^ In another study, *Lactobacillus* levels were reproducibly elevated upon ADF only in mice fed with normal chow, whereas the genus *Allobaculum* was identified as exclusively enriched in mice undergoing ADF and fed with high‐fat diets.^121^ Interestingly, the *Allobaculum* genus is an active glucose metabolizer that produces butyrate and lactate.^121^, ^122^ These findings suggest that ADF induced production of acetate and lactate^118^ by various species of the intestinal bacteria. Yet, more studies are needed to define the effect of lactate and SCFAs on WAT browning and on the host thermogenesis and energy expenditure.

**FIGURE 1:**
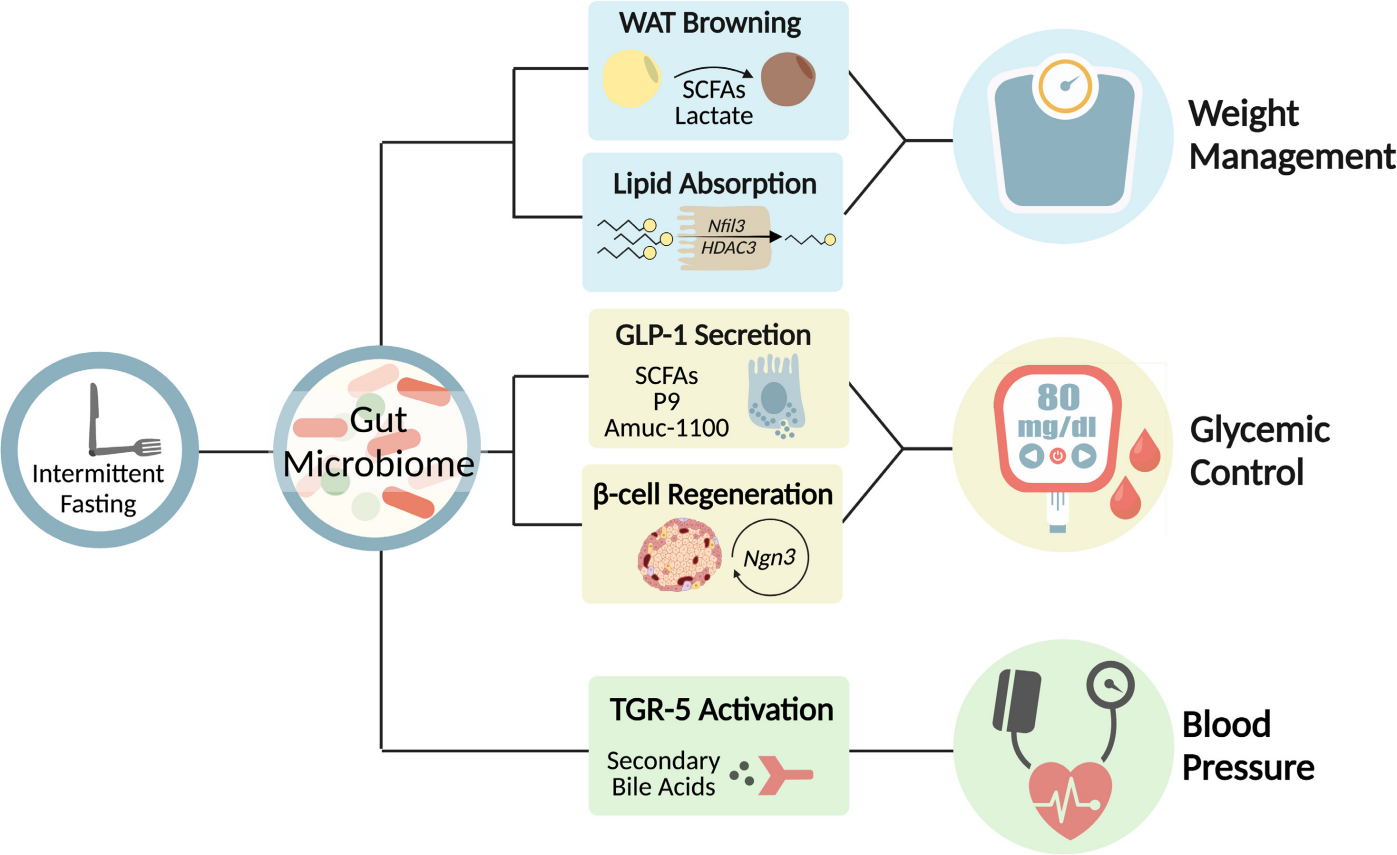
Intermittent fasting mediated changes in the gut microbiome composition and function, which in turn may affect cardiometabolic health. Gut microbiome driven WAT browning and lipid absorption contribute to weight management. Microbiome effects on glycemic control through GLP‐1 secretion and β‐cell regeneration. Microbiome derived secondary bile acids activates TGR‐5 to control blood pressure. GLP‐1, glucagon‐like peptide‐1; HDAC3, histone deacetylase 3; Nfil3, nuclear factor interleukin‐3; Ngn3, Neurogenin‐3; SCFA, short chain fatty acids; TGR‐5, Takeda G protein‐coupled receptor 5; WAT, white adipocyte tissue. Figure created with BioRender (biorender.com)

In addition to adipocyte thermogenesis, alterations in the gut microbiome may influence lipid uptake during timely eating. There are several mechanisms by which this may occur, including regulation of the **nuclear factor interleukin‐3 (NFIL3)**, a transcription factor that is controlled by the circadian clock and regulates the rhythmic expression of genes involved in the uptake, processing, and storage of lipids in intestinal epithelial cells.^103^ The rhythmic oscillations in NFIL3 are driven by the gut microbiome through activation of innate immune cells response.^103^ A second factor controlling host circadian lipid uptake is **histone deacetylase 3 (HDAC3)**.^104^ Stimulation of rhythmic expression and recruitment of HDAC3 to the chromatin results in synchronized circadian oscillations of histone acetylation in the gut epithelium, which in turn regulate gene expression of nutrient transporters, thus affecting nutrient uptake and lipid absorption.^104^ However, the interplay between feeding rhythms, gut microbiome oscillations, NFIL3, HDAC3, and body weight control has not been fully elucidated to date. Collectively, the gut microbiome influences energy metabolism by regulating genes that control the uptake of lipids and nutrients and by production of microbial metabolites affecting adipose tissue browning (Figure 1).

***Glycemic control*.** Studies in rodents and monkeys demonstrated consistent beneficial effects of IF on glycemic control.^5^, ^123^ Several human studies suggest that IF diets can lower blood glucose and may lead to better glycemic control in T2D patients.^11^, ^18^, ^124^ However, recent randomized controlled trials did not provide evidence that IF diets are more effective in regulating the glycemic response compared with matched caloric restriction in lean,^20^ overweight, and obese individuals^21^, ^22^, ^26^, ^125^ and T2D patients.^126^ The gut microbiome composition correlates with blood glucose levels^67^, ^68^ and adapts to fasting and refeeding periods in daily life, including circadian eating patterns and TRF (eg, as part of religious fasting).^91^, ^127^ In mice, the genus *Lactobacillus* is highly abundant during fasting, whereas the species *Akkermansia muciniphila* is highly enriched during feeding.^72^, ^128^ Apart from diurnal variations, *A. muciniphila* is significantly expanded in both humans and mice undergoing TRF.^76^, ^91^ These compositional changes are intriguing as *A. muciniphila* is inversely correlated with blood glucose levels in rodents and humans.^129^ Furthermore, an increase in *A. muciniphila* is associated with enhanced secretion of GLP‐1, an insulinotropic hormone secreted by enteroendocrine L cells in response to a meal and plays a key role in whole‐body glycemic control.^129^ GLP‐1 secretion follows a circadian rhythm, with higher levels of postprandial GLP‐1 following glucose loads at the active phase and lower levels during the resting phase,^130^, ^131^, ^132^, ^133^ and is additionally regulated by the clock machinery in L cells.^134^ Martchenko et al^128^ found that the fluctuating secretion of GLP‐1 was attenuated in obese mice fed with western diet and in microbiome‐depleted mice. This study demonstrated that restoration of GLP‐1 circadian rhythmicity in germ‐free mice was achieved by transfer of fecal microbiome from conventional mice consuming normal chow diet into obese mice. These results suggest that diurnal changes in the microbiome may play a central role in the circadian secretion of GLP‐1 and its subsequent effects on glucose homeostasis.^128^ As for mechanisms, recent studies have identified several putative biomolecules produced by *A. muciniphila* that could trigger the secretion of GLP‐1 from intestinal L cells. Among them, propionate and the proteins P9 and Amuc‐1100 may stimulate GLP‐1 secretion from L cells.^135^ It remains unclear how these bacterial molecules control GLP‐1 secretion, by which mechanisms and physiological conditions, and whether microbial modulation of GLP‐1 is driven by IF diets.

IF or FMD given to obese and hyperglycemic mice, or diabetic ***db/db* mice** lacking the leptin receptor, led to improved glycemic control and amelioration of T2D, respectively.^136^, ^137^, ^138^ One of the primary underlying mechanisms for the beneficial effect of FMD on glucose homeostasis includes activation of **Neurogenin 3 (Ngn3)**, a transcription factor that is essential for the development of insulin‐producing β cells.^136^ Wei et al^138^ confirmed that the regeneration of β cells occurs upon exposure to intermittent FMD and showed that it follows a restructuring of the gut microbiome, which was correlated with blood glucose levels in *db/db* mice. According to 16S rRNA sequencing of stool microbiome, FMD increased the relative abundance of *Parabacteroides distasonis* and *Blautia*, and decreased the abundance of *Lachnospiraceae NK4A136*, *Prevotellaceae*, *Alistipes*, and *Ruminococcaceae*, which correlated with lower blood glucose levels.^138^ In this context, *Blautia*, which had higher abundance in FMD,^138^ was found to be relatively increased after T2D medication treatment in diabetic rats.^139^ However, a direct causal effect of the gut microbiome on β‐cell proliferation during IF diets has not been defined and merits further investigation. It is noteworthy that the induction of such phenotypes does not necessarily require complete fasting days, because ADF can be combined with alternate days of a leucine‐free diet in order to slow the progression of T2D.^140^

Collectively, influences of nutritional timing on the gut microbiome and on GLP‐1 secretion as well as on β‐cell proliferation may contribute to improved glycemic control and insulin sensitivity and thereby provide a rationale for diet and microbiome‐based therapeutic potential in management of T2D (Figure 1). In agreement with the effect of IF diets on β cells regeneration in rodents, human randomized clinical trials suggested a superior effect of periodic fasting (5:2 diet) on fasting insulin compared to a matched group with daily caloric restriction.^23^, ^25^, ^125^

***Blood pressure*.** Hypertension refers to persistently high arterial blood pressure that, if left untreated, can lead to a number of devastating consequences such as heart failure and peripheral vascular disease.^141^ Although no cure for primary hypertension is currently available, hypertension is controllable by chronic antihypertensive medical treatment coupled with lifestyle modifications.^142^ There are several indications that IF and meal timing may contribute to improved hypertension in mice and humans.^11^, ^18^, ^143^, ^144^, ^145^ With these observations notwithstanding, the validity of IF on blood pressure has been questioned in a number of randomized controlled studies with large numbers of participants, showing that IF, ADF, or 5:2 diet have no additive effect on blood pressure.^21^, ^23^, ^25^ Although the effectiveness of IF is debatable, the DASH diet, which stands for Dietary Approaches to Stop Hypertension, offers a low sodium diet that may reduce hypertension.^146^, ^147^ A recent study suggested that a five‐day fast followed by a modified DASH diet reduces systolic blood pressure in patients with hypertensive metabolic syndrome.^147^ This fasting regime also altered the gut microbiome, including several bacterial communities and genes associated with SCFA production.^147^ Within the IF group in this study, a prediction of sustained systolic blood pressure response was performed using machine‐learning analysis of baseline microbiome data, identifying *Desulfovibrionaceae*, *Hydrogenoanaerobacterium*, *Akkermansia*, and *Ruminococcaceae* as potential contributors to controlled hypertension.^147^ In rats, five weeks of ADF regime significantly reduced blood pressure in hypertensive stroke‐prone animals.^69^ This phenotypic change was accompanied by altered microbiome configuration including elevated *Bacteroides uniformis*, *Lactobacillus reuteri*, *Lactobacillus johnsonii*. Mechanistically, the ADF diet was associated with a microbial shift toward bacteria producing secondary bile acids, both conjugated and unconjugated (such as taurolithocholic acid, taurodeoxycholic acid, **tauroursodeoxycholic acid** **[** **TUDCA]**, LCA, glycochenodeoxycholic acid, and more), and activation of the bile acid receptor **Takeda G protein‐coupled receptor 5** (**TGR5**; Figure 1). Moreover, treatment of these hypertensive rats with cholic acid or TGR5 agonist attenuated elevated blood pressure, thus exceeding the need for ADF.^69^ Fecal transplantation from ADF‐fed rats to germ‐free rats prevented elevated systolic blood pressure, showing the causal role of the gut microbiome in lowering blood pressure.^69^ Further studies need to define the target tissue and cell types that respond to the secondary bile acids as well as the cellular pathways induced by these bile acids, leading to the reduction in blood pressure. In addition, more trials are required to define the efficacy and sustainability of different IF diets in lowering blood pressure in patients with various cardiometabolic derangements.

## 3 INTERMITTENT FASTING AND MICROBIAL REGULATION IN LONG‐TERM CARDIOMETABOLIC COMPLICATIONS

**FIGURE 2:**
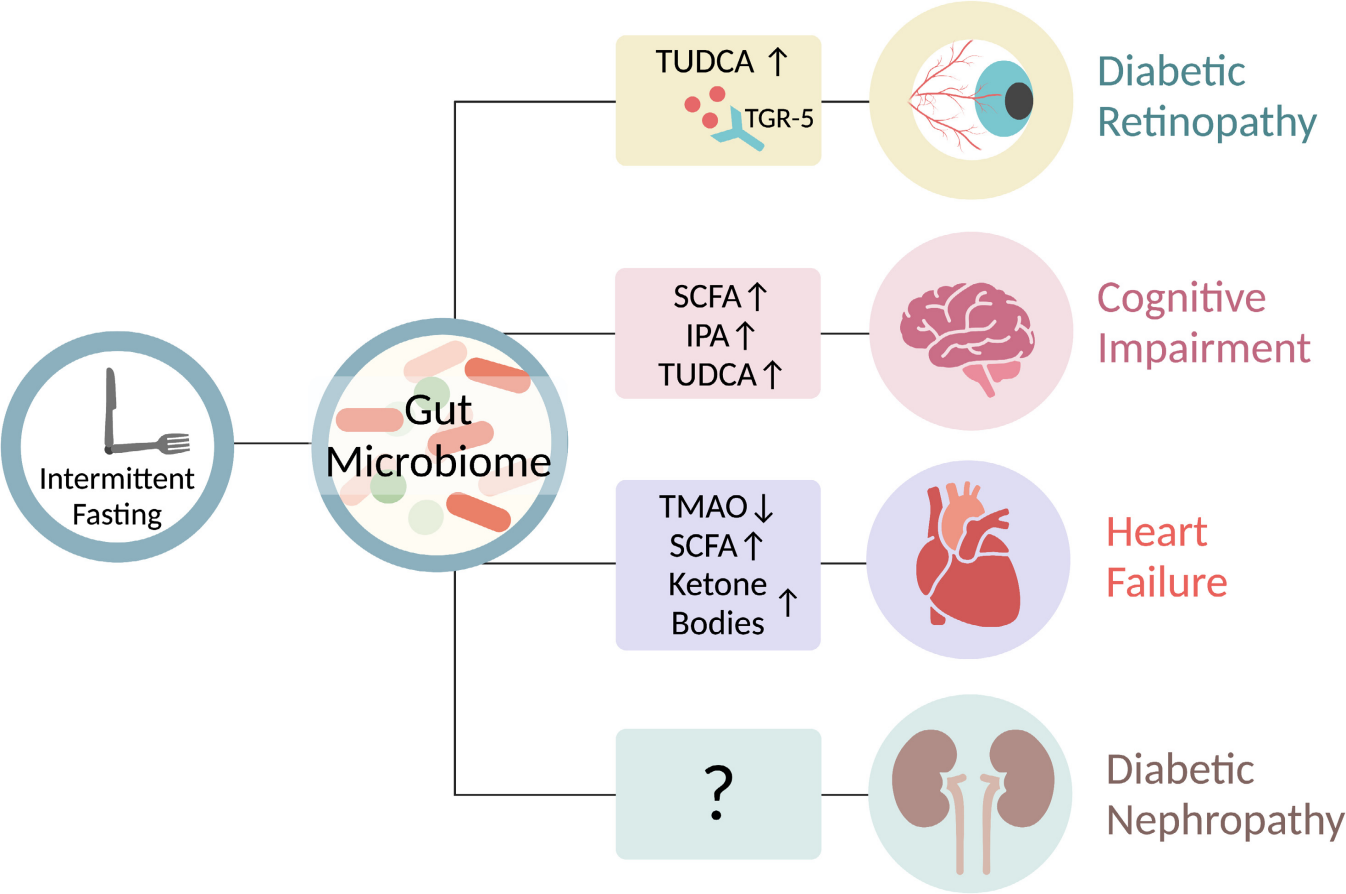
Intermittent fasting mediated gut microbial metabolites may affect cardiometabolic complications. IPA, Indole‐3‐propionic acid; SCFA, short chain fatty acids; TGR‐5, Takeda G protein‐coupled receptor 5; TMAO, trimethylamine N‐oxide; TUDCA, tauroursodeoxycholic acid. Figure created with BioRender (biorender.com)

CMDs are progressive diseases, demonstrating long‐term and devastating consequences. A common manifestation in T2D patients is microvascular complications that include retinopathy and nephropathy as well as macrovascular diseases such as CVDs. IF diets, as part of nutrition therapy, have emerged as a potential intervention for managing several long‐term complications of T2D, including retinopathy, cognitive decline, heart failure, and nephropathy (Figure 2).

***Retinopathy*.** Diabetic retinopathy is a complication of T2D that affects the blood vessels of the retina and can lead to blindness in untreated individuals.^148^ Fortunately, the risk of vision loss due to diabetic retinopathy can be reduced by early detection and timely treatment to control glucose levels and blood pressure.^149^, ^150^ It has been suggested that the gut microbiome may be involved in the pathogenesis of diabetic retinopathy.^85^, ^151^, ^152^, ^153^, ^154^, ^155^ According to Das et al,^152^ the gut microbiome of patients with diabetic retinopathy differs significantly from healthy individuals as well as from T2D patients without retinopathy. Examples of these differences include decreased abundance of *Bifidobacterium* and *Turicibacter*, and increased abundance of *Akkermansia* in diabetic retinopathy.^152^ Following this observational study, Huang et al^154^ presented the potential use of the gut microbiome as a discriminating biomarker of diabetic retinopathy and found that *Pasteurellaceae*, *Oxalobacteraceae*, and *Gallionellaceae* bacterial families were the main biomarkers distinguishing patients with T2D and patients with diabetic retinopathy, which could be helpful for diagnosis of retinopathy. Among them, the bacterial family *Pasteurellaceae* was specifically reduced in T2D patients with retinopathy and loss of this bacteria can serve as a predictive biomarker for the disease.^154^ Surprisingly, Das et al^152^ did not report similar changes. In relation to the IF diet regimes, a study by Beli et al^85^ reported an association between the gut microbiome, dietary patterns, and diabetic retinopathy. The authors use diabetic (*db/db*) mice to compare the classical markers of diabetic retinopathy in mice fed with an ADF diet compared to those fed ad libitum. They found that the fasting regime prevented the increase in the number of acellular retinal capillaries and reduced the infiltration of inflammatory cells into the retina.^85^ Diabetic mice fed with ADF featured a significant expansion of Firmicutes phylum, and more specifically at the genus level, enhanced abundance of *Oscillospira*, *Ruminococcus*, and *Turicibacter* and reduced abundance of *Bacteroides*, *Akkermansia*, *Bifidobacterium*, and *Allobaculum*.^85^ Along with the changes in gut microbiome composition, the authors observed that diabetic mice exhibited differences in diurnal microbial patterns compared to nondiabetic mice, which also altered in response to ADF.^85^ Although the functional significance of the microbial oscitations following the ADF diet has not been addressed in depth, this observation is important as it implies that the effect of IF diet is highly dependent on the health status of the host. Notably, ADF enhanced the metabolism of primary to secondary bile acids, such as TUDCA, only in diabetic mice. The receptor for TUDCA, TGR5, is expressed in retinal ganglion cells, suggesting that microbial production of TUDCA can potentially affect the retina. Supplementation of diabetic mice with a potent agonist for TGR5 resulted in diminished diabetic retinopathy, featured by reduced retinal inflammation and lower acellular capillaries. Consequently, it is feasible that ADF may lead to altered gut bacterial production of secondary bile acids, activation of retinal TGR5, and protection against retinal degeneration^85^ (Figure 2). Sor far, it is unknown which cells in the retina respond to the secondary bile acids and the signaling pathways induced by the bile acids resulting in protection against retinal degeneration. Taken together, these results suggest that IF interventions such as ADF may lead to a unique modification of the gut microbial communities and metabolites that may contribute to retinopathy diagnosis and may potentially ameliorate diabetic retinopathy.

***Cognitive impairment*.** T2D can cause pronounced central nervous system complications, including structural alterations or brain atrophy, cerebral microvascular damage, neuroinflammation as well as changes in electrophysiological properties of the brain that ultimately result in cognitive deficits.^156^, ^157^ These alterations in cognition and brain structure may, over time, lead to an acceleration of cognitive decline and an increased risk for age‐related neurodegenerations such as Alzheimer's disease.^158^ Multiple factors were proposed to induce cognitive impairments in diabetes, these include brain insulin resistance and lower glucose uptake as well as, disrupted neurotransmitter metabolism.^156^, ^159^ In animal models, various types of IF diets have been reported to benefit brain health and delay the development of neurodegenerative diseases.^160^, ^161^, ^162^ Notably, the gut microbiome may play a role in modulating cognitive functions induced by IF.^80^, ^86^, ^163^, ^164^ Liu et al show a link between the gut microbiome and cognitive functions during ADF treatment of diabetic *db/db* mice.^86^ These diabetic mice exhibited cognitive decline, but a 28‐day ADF regimen improved anxious behavior, locomotor activity, and synapse structure, along with preservation of insulin signaling and mitochondrial biogenesis in the hippocampus. The improvement in brain functions of the *db/db*‐ADF mice group was accompanied by an increased abundance of genus *Lactobacillus* and *Odoribacter* and decreased *Enterococcus*, *Streptococcus*, and *Enterococcaceae*. The protective effect of ADF on the cognitive functions of the diabetic mice was partly abolished upon antibiotic treatment. In order to determine how these microbes might affect cognitive function, the authors used genome‐based functional predictive analysis and metabolomics.^86^ They found enrichment of primary and secondary bile acid biosynthesis pathways in the ADF group. In addition, *db/db*‐ADF mice group exhibited an increased fecal and plasma levels of several microbial‐related metabolites, among these metabolites are SCFAs, TUDCA (secondary bile acid that was also protective against retinopathy), **indole‐3‐propionic acid (IPA)**, and serotonin. Supplementation of these metabolites improved cognitive functions and insulin sensitivity in *db/db* mice.^86^ The signaling pathways induced by the microbial metabolites, the connection to brain insulin sensitivity, and the responding cells in the brain remain to be determined. Collectively, these studies propose that gut bacterial species and metabolites induced by ADF may contribute to alleviation of diabetes‐induced cognitive impairment (Figure 2) and suggest that the bacterial metabolites may modulate features of brain function even in the absence of ADF.

***Heart failure*.** Murine models of insulin resistance have suggested that IF diets such as TRF^12^, ^137^ and FMD^138^ may lead to improvement of hypertension, dyslipidemia, hyperglycemia, and hyperinsulinemia, all constituting general indicators of cardiovascular health.^165^ Initially, clinical studies suggested that IF diets, including ADF^21^ and TRF,^166^ improve cardiac protection in T2D patients. A number of human randomized controlled trials have reported that restricting calories and IF diets are equally effective in lowering hypertension, dyslipidemia, hyperglycemia, and hyperinsulinemia, all of which are risk factors for cardiac events.^20^, ^21^, ^22^, ^23^, ^25^, ^26^ Several studies have shown a compositional alterations of gut microbial communities in patients with heart failure.^167^, ^168^, ^169^ A study in Dahl salt‐sensitive rats, which develop hypertension and are more prone to heart failure, showed that supplementation with the probiotic bacteria *Lactobacillus plantarum* 299v reduced their susceptibility to heart failure and resulted in better recovery following myocardial infarction.^170^ Several mechanisms have been proposed to explain the potential link between the gut microbiome and heart failure, including microbiome‐induced modulation of inflammation, intestinal permeability, and associations made to bacterial overgrowth and bacterial biofilm formation.^167^, ^168^, ^169^ Expansion of pathogenic bacteria was also detected in patients with heart failure in several studies.^169^, ^171^ A study by Crawford et al^172^ sheds light on how IF may benefit the heart, proposing that the gut microbiome can produce SCFAs that provide the heart with adequate energy during fasting. Their study showed that germ‐free mice compared to conventionalized mice exhibited reduced liver production of ketone bodies. According to this study, fasting was associated with higher abundance of *Bacteroides* that may be responsible for the production of SCFAs, especially acetate, which may be utilized for hepatic ketogenesis, thereby providing a source of energy for the heart.^172^ During fasting, the heart of germ‐free mice utilized glucose instead of ketone bodies, which was reverted to ketone body metabolism by feeding a ketogenic diet. Therefore, it is plausible that the beneficial effects of fasting on cardiac function depends, at least in part, on the presence and the ability of intestinal bacteria to enhance the generation of ketone bodies that can be oxidized by the heart^172^ (Figure 2). A recent study by Mistry et al^173^ suggested that the circadian clock governs the bacterial effects on the heart, including those reflected during TRF. This study reported that mice undergoing myocardial infarction and treated with antibiotics had an increased incidence of heart failure, which was abrogated by cohousing with mice without microbiome depletion. Interestingly, mice with a disrupted circadian rhythm showed perturbation in gut microbiome rhythmicity, as well as impaired ischemic cardiac healing and heart failure, even in the presence of an intact microbiome.^173^ With respect to IF dietary patterns, the authors used TRF diet and showed that mice fed only at the dark phase before myocardial infarction had improved cardiac function compared to mice fed only at the light phase. Thus, both the gut microbiome and TRF can individually affect heart failure outcomes.^173^ However, it remains to be determined whether the gut microbiome mediates the improved cardiac functions induced by TRF.

An additional mechanism by which the gut microbiome can impact cardiovascular health is through the gut bacterial production of **trimethylamine‐*N*‐oxide (TMAO)**. A seminal study by Wang et al^174^ showed that gut microbial metabolism of dietary choline and L‐carnitine resulted in production of TMA, which was converted by the host liver to TMAO. The TMA‐TMAO pathway was found to be involved in atherosclerosis, platelet hyperactivity, and thrombosis and was used to predict an increased risk of CVDs.^174^, ^175^ Fasting affects liver metabolism and is associated with decreased TMAO^176^ (Figure 2). It has been previously suggested that maintaining low levels of TMAO may be particularly helpful in preventing T2D‐associated cardiomyopathy.^177^ Recently, elevated TMAO levels were found in *db/db* mice during both daytime and nighttime, which was associated with loss of diurnal oscillations of various gut bacteria.^113^ The authors suggested that it may be possible to restore the lost diurnal bacterial oscillations by restricting the feeding during the active period.^113^ In accordance with findings in rodents, it remains to be determined whether IF diets can harness the gut microbiome, regulate TMAO levels and lead to effective improvements in cardiovascular outcomes in T2D patients.

***Nephropathy*.** Kidney dysfunction or nephropathy as a result of diabetes is the leading cause of chronic kidney disease.^178^ In diabetic patients, poor glycemic control and hypertension can lead to glomerular hyperfiltration, albuminuria, nephrotic proteinuria, and development of end‐stage renal disease.^179^, ^180^, ^181^ Several studies have shown alterations in gut bacterial abundance in patients with diabetic nephropathy compared to controls, and overall lower bacterial diversity was correlated with disease progression.^182^, ^183^, ^184^ In rodents, diabetic nephropathy was associated with altered bacterial communities and microbial metabolites like phenyl sulfate^185^, ^186^ and correlated with activation of the renin‐angiotensin system.^187^, ^188^ Several observational studies have tested the effect of Ramadan fasting on diabetic nephropathy severity, but none of them showed significant changes in renal functions. These reports were not randomized controlled studies, had small sample sizes, and contained patients with different medications or dialysis treatment. Furthermore, most of the studies were conducted in countries where fasting durations were 12‐14 h during the winter season and thus cannot be generalized to longer fasting durations.^189^ Currently, the efficacy of IF on kidney functions and diabetic nephropathy and the involvement of the gut microbiome in this process have not been elucidated.

## 4 CHALLENGES AND LIMITATIONS

Recent studies report that during multiple IF diets, the intestinal microbiome may act as a signaling hub driving the improvement in cardiometabolic complications. However, there are several technical and conceptual obstacles that still need to be carefully taken into consideration in interpreting these studies. First, IF diets regimens are inconsistently defined across studies and the long‐term effects of IF diets were mostly undetermined. From the perspective of microbiome analyses, some studies are lacking a suitable availability of some microbial datasets and resources. Furthermore, microbiome sampling in most of the human studies analyze fecal samples and disregard regional differences in the gut microbiome composition and function along the gastrointestinal tract. Additionally, some microbiome studies feature technical differences in data acquisition and analysis modalities (eg, sample allocation, extraction, or sequencing methods) differ between studies, making comparisons between their results challenging. There is also a global inability to generalize conclusions on microbiome structural alterations across human studies, due to differences between ethnical, geographical and demographical groups and variability in body mass index, sex, age, physical activity, and other clinical parameters of the participants in different studies. In addition to technical and human individual variabilities, other differences derived from personalized uniqueness of microbiome configurations constitute a formidable challenge in generalizing such results. However, the individualized microbiome heterogeneity is not necessarily a disadvantage, because it may allow for identification and development of personalized responses to various IF interventions and forms a basis (together with a variety of host clinical and laboratory features) for personalized nutrition.

## 5 FUTURE PERSPECTIVES

The discovery that the gut microbiome may modulate the metabolic outcomes of IF diets may provide mechanistic explanations for the observed phenotypes as well as for the personal response to the IF regimens observed in different individuals exposed to a similar dietary intervention. Indeed, gut microbiome composition and function differ between humans and rodents following IF diets as compared to those following continuous food consumption. In addition to the altered bacterial configuration, IF collectively leads to bacterial production of specific metabolites such as secondary bile acids, SCFAs, and ketone bodies, which may have a distinct downstream impact on host physiology and disease. Although we highlight in this review how changes in microbial configuration and metabolite production may potentially influence the clinical outcomes of different cardiometabolic abnormalities, comprehensive future research is warranted to decipher the molecular and chemical processes that mediate these effects. In addition, further research is needed to address the effect of the microbiome on additional organs such as the lung, skin, muscles, and kidneys, whose functions could also be influenced by IF diets.^190^, ^191^, ^192^, ^193^ Moreover, it remains to be determiced what is the role of the IF‐microbiome axis in several T2D‐related complications, such as the susceptibility to fungal infections,^194^ diabetic foot ulcers, and diabetic nephropathy, which account for high morbidity and mortality rates in T2D patients.^195^ Another question is whether a combination of IF with a defined diet composition or food choice consumed in eating days (eg, the aforementioned 5:2 DASH diet^147^) can provide targeted benefits for certain CMD‐related conditions. In this respect, it is worth noting that although the nutrient composition of the diet has a tremendous effect on the gut microbiome,^196^ the impact on CMD has not been elucidated. Other factors such as digestibility and transit time can be modulated during multiple IF diets. However, their relevance to CMD is unclear.

A major challenge in translating IF regimens into clinical practice is the contradictory results found in human studies investigating the effects of IF diets on human metabolism.^22^, ^23^, ^24^, ^25^, ^26^, ^197^ One reason for these discrepancies stems from the human individual response to IF diets. For example, Berry et al^198^ showed that postprandial responses to food vary across individuals when the same meals are given at different hours. Because the gut microbiome is involved in the personal response to food,^70^ it would be reasonable to assume that the personal response to IF can also be linked to the gut microbiome. Collectively, more well‐controlled, prospective, longitudinal clinical studies are required to define the microbial alterations in different IF diets that can be further translated into clinical practice.

The idea that the gut microbiome is affected by IF diets and affects normal host physiology and CMD progression allows using these findings as a potential basis for therapeutic intervention. Identification of bioactive microbial‐derived molecules potentially affecting human metabolism can be developed as a bona fide “postbiotic therapy,” bypassing the high interindividual variability of the microbiomes producing such molecules. One such example is SCFAs, which in rodents demonstrated beneficial effects on adipose tissue browning and energy expenditure,^118^ secretion of GLP‐1 from L cells and glycemic control,^135^ and cardiac functions.^172^ Secondary bile acids are another example of bacterial molecules that showed beneficial cardiometabolic effects in mice, as exemplified by secondary bile acids that attenuated hypertension^69^ and show protection against diabetic retinopathy^85^ and cognitive impairments.^86^ On the other hand, specific microbial products can induce worsened metabolic outcomes, such as TMAO that was involved in cardiac dysfunctions and associated with a higher risk for CVDs.^174^, ^175^ In many cases, these metabolites were modified during implementation of different IF diets (Figure 2) and contributed to the effect of IF on metabolic outcomes. An addition of the secondary bile acid TUDCA or an agonist of the bile acids receptor TGR5 led to protection from cognitive decline and retinopathy in diabetic mice, bypassing the need for IF diet,^85^, ^86^ whereas microbial enzyme inhibitors for TMAO^169^, ^199^ may alleviate cardiometabolic derangements. These and other microbiome‐based interventions merit further studies.

## CONFLICT OF INTEREST

E.E. is a scientific founder of DayTwo and BiomX, and a paid consultant to Hello Inside GmbH. The remaining authors declare no competing interests.
